# Inadvertent Intraoperative Defibrillation Secondary to Electrocautery Grounding Pad Placement

**DOI:** 10.7759/cureus.29391

**Published:** 2022-09-21

**Authors:** Joseph A McGuire, Jeremiah Hayanga, Charles Barry, Anna Carpenter, Benjamin Frye, James Hughes, David Schwartzman, Heather Hayanga

**Affiliations:** 1 Department of Anesthesiology, West Virginia University School of Medicine, Morgantown, USA; 2 Department of Cardiovascular and Thoracic Surgery, West Virginia University School of Medicine, Morgantown, USA; 3 Department of Orthopaedics, West Virginia University School of Medicine, Morgantown, USA

**Keywords:** electrocautery, cardiovascular implantable electronic device, automatic cardioverter defibrillator, peri-operative, electromagnetic phenomena, implantable defibrillators

## Abstract

Intraoperative defibrillation secondary to the usage of electrocautery in a patient with a cardiovascular implantable electronic device is a rare occurrence, and below-the-umbilicus electrocautery use causing inadvertent defibrillation is a near-zero risk. Defibrillation secondary to electrodispersive pad (EDP) radiofrequency dispersion has only ever been theorized. In this report, we describe the case of a 67-year-old male with an automatic implantable cardioverter defibrillator (AICD) undergoing a robotic-assisted left anterior total hip arthroplasty for left hip osteoarthritis who experienced inadvertent intraoperative defibrillation concurrent with electrocautery usage. The defibrillations ceased following contralateral EDP repositioning and application of a donut magnet overlying the patient’s AICD.

## Introduction

As medical technology has continued to advance and improve patient welfare, there has been an increase in the overall usage of cardiovascular implantable electronic devices (CIED), including automatic implantable cardioverter defibrillators (AICD) [1]. Additionally, electrocautery devices - particularly monopolar devices - have become a centerpiece of the modern surgical armamentarium [2]. The interaction between CIEDs and electrocautery devices has been widely studied, with the earliest reported literature on the subject dating back to 1999 [3]. Studies have shown that, while electrocautery applied above the umbilicus may cause inadvertent defibrillation of the AICD, below-the-umbilicus electrocautery usage poses a near-zero risk of causing clinically noticeable AICD activity [4,5].

The 2021 European Society of Cardiology (ESC) guidelines on cardiac pacing and resynchronization therapy offer vague advice regarding perioperative alteration of CIEDs for surgeries utilizing below-the-umbilicus electrocautery [6]. The Heart Rhythm Society (HRS)/ASA expert consensus statement for perioperative CIED management published in 2011 recommends either not altering the AICD preoperatively or applying a prophylactic magnet for procedures below the umbilicus [7]. Current American Society of Anesthesiologists (ASA) practice advisory recommendations regarding AICD management in surgeries superior to the umbilicus exist; however, those for surgeries inferior to the umbilicus are less clear [8].

Thus, we report a case of an elderly gentleman who had inadvertent intraoperative AICD defibrillation thought to be secondary to electromagnetic stimulation due to the electrodispersive pad (EDP) position while undergoing surgery below the umbilicus.

## Case presentation

A 67-year-old male presented for robotic-assisted left anterior total hip arthroplasty to correct osteoarthritis of the left anterior hip following the failure of conservative treatment. The patient weighed 107 kg (235 lbs) and was 1.753 m (5’9”) tall. Aside from the patient’s degenerative joint disease, his past medical history consisted of hyperlipidemia, hypothyroidism, obstructive sleep apnea, gastroesophageal reflux disease with a history of antral ulcer, atrial fibrillation, ventricular tachycardia, and non-ischemic cardiomyopathy status post AICD placement. Other procedures include a gastric bypass, tonsillectomy, and ventricular tachycardia ablation. The patient’s AICD was placed 19 years earlier due to cardiac arrest secondary to ventricular tachycardia and non-ischemic cardiomyopathy with a left ventricular ejection fraction of 30-35%. The patient’s most recent transthoracic echocardiogram demonstrated a 40-45% ejection fraction with evidence of moderate mitral and tricuspid regurgitation. Preoperative laboratory analysis demonstrated no abnormalities in serum electrolytes, lipids, or glucose levels. The AICD had been interrogated within six months of the elective surgery; there were no recent defibrillations, the patient was not pacemaker dependent, and an estimated 6.3 years of battery life remained.

**Figure 1:**
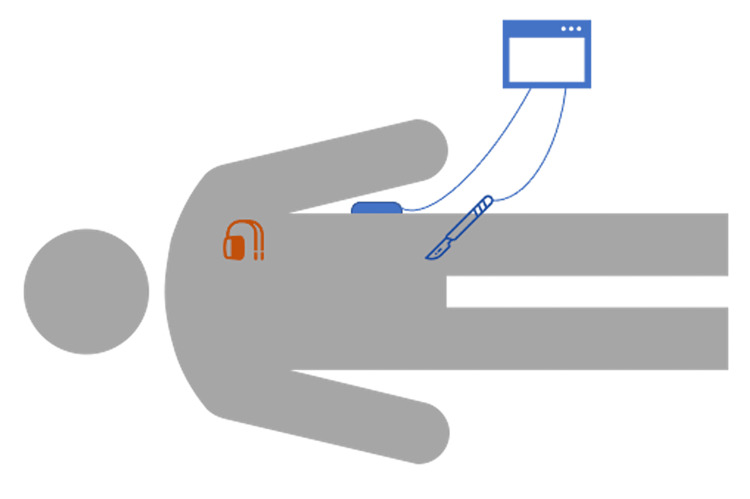
Electrodispersive Pad Placement in Patient with Automatic Implantable Cardioverter Defibrillator.

On the day of surgery, a spinal block with monitored anesthesia care was planned for anesthetic management. The spinal block was completed using 2mL of 0.75% hyperbaric bupivacaine injected intrathecally. The patient was placed in a supine position and the EDP was placed on the patient’s left flank (Figure 1). After induction of anesthesia with a propofol infusion, both a nasal and oral airway was inserted to allow for adequate patency of airways, considering the patient’s obstructive sleep apnea history. During the operation, the patient's AICD defibrillated on two separate occasions that were both concurrent with monopolar electrocautery usage. The surgical procedure was paused, and the EDP was repositioned from the left flank to the right flank. A magnet was placed over the AICD generator to inhibit its antitachycardia therapy functionality. The procedure was then resumed and completed with no additional complications. Postoperatively, a 12-lead ECG was ordered, and the electrophysiology team was consulted. The consultants found that the AICD had no abnormalities or malfunctions, and the inadvertent intraoperative defibrillations were confirmed. The patient recovered well postoperatively and suffered no clinically detectable myocardial damage.

## Discussion

Monopolar electrocautery instruments are also known as “active electrodes” when energized. To carry out the functions of tissue coagulation or fulguration, a second node known as the “dispersive electrode” is required. The dispersive electrode, or EDP, functions to distribute the energy from the active electrode to prevent thermal injury to the patient [9]. This does not pose a significant risk in most patients, and even most patients with CIEDs. However, any superfluous energy has the potential to interact with and lead to the malfunctioning of CIEDs.

Placement of the EDP is perhaps the most important component of ensuring the safe usage of an electrocautery device. Several incidences of intraoperative thermal injuries occurring secondary to improper placement or dysfunction of the EDP have been previously reported [10-13]. Methods of reducing the likelihood of this complication include positioning the pad as close to the operative site as possible over dry, shaven, and well-vascularized surfaces with maximal surface area to allow for adequate diffusion of electrical energy. It is recommended that bony prominences, adipose tissue, scar tissue, skin over implanted metal prostheses, hairy surfaces, and pressure points be avoided [9]. It may be placed on the distal extremities such as the patient’s calf or forearm, however, these aren’t preferred locations due to there being less surface area for adequate diffusion of electrical energy. No specific guidelines exist that indicate best practices for alternative EDP placement in various surgical scenarios, such as the one encountered in this report.

While EDP placement has been shown to induce thermal injury with improper placement or device dysfunction, inadvertent defibrillation secondary to EDP placement has never been reported. In fact, the only time defibrillation secondary to below-the-umbilicus monopolar electrocautery usage has ever been reported to occur was in a patient undergoing total knee arthroplasty [14]. The authors did not provide a theory on whether the electrical energy dispersed by the EDP on the patient’s left thigh or the energy generated by the cautery device itself was responsible for the inadvertent defibrillations. Although the event was not directly attributed to EDP placement, the authors did warn against the usage of pads that call for placement of the EDP on the operating table due to increased surface area for the electric field to be generated.

Neither the 2011 HRS/ASA expert consensus statement for perioperative CIED management nor the 2021 ESC guidelines on cardiac pacing and cardiac resynchronization therapy offer recommendations for proper placement of the EDP. The 2020 ASA Practice Advisory for perioperative CIED management, which is developed from a survey of expert consultant anesthesiologists and ASA members, has no clear recommendations regarding below-the-umbilicus electrocautery use [8]. In these guidelines, when monopolar electrosurgery use is planned as inferior to the umbilicus, consultants were equivocal (34% strongly agree, 25% equivocal, 34% strongly disagree) and ASA members disagreed (40% disagree) with the recommendation to alter the pacing function of a CIED to asynchronous pacing in a pacing-dependent patient. Moreover, consultants agreed (23% strongly agree, 29% agree), and ASA members were equivocal (22% agree, 24% equivocal, 31% disagree) with the recommendation to suspend an AICD’s antitachycardia function [8]. This lack of consistency requires that the anesthesiologist independently assess the clinical situation and determine the best plan of action for the individual patient. Furthermore, the 2020 ASA practice advisory guidelines for perioperative CIED management recommend placing the EDP such that the current does not pass through or near the CIED generator or the leads. This may be challenging, though, depending on the surgery and patient position. In our patient, the EDP was placed initially on the left flank, so as to adhere most closely to standard guidelines. Given the patient’s height and assumed position of the AICD and its respective leads, the pad was initially thought to be positioned in a proper location.

The 2021 ESC guidelines on cardiac pacing and cardiac resynchronization therapy recommend tailoring the peri-operative strategy on alteration of the CIED based on the needs and values of the patient, procedure, and device. However, it is mentioned in the guidelines that most procedures will not require intervention and electromagnetic interference with CIEDs by electrocautery >5cm from the CIED and monopolar electrocautery below the umbilicus is a rare event. In contrast, an editorial published in 2019 called for the preoperative alteration of the AICD, whether by disabling the device or preemptively placing a donut magnet, despite the surgical site of the procedure, due to the potential risk of inadvertent defibrillation and subsequent myocardial injury [15]. A separate original article describing potential interactions and surgical best practices when concurrently using electrocautery and CIEDs warned of the usage of monopolar electrocautery and suggested instead utilizing bipolar cautery or deactivating the CIED for the duration of the case altogether [3]. While both suggestions have merit, both present potential drawbacks to the surgical and anesthesia teams in terms of efficiency, turnover time, and the readily available access to electrophysiology services. Furthermore, drawbacks may include differences in the ability to cut and coagulate with bipolar electrocautery when compared to monopolar electrocautery and the potential complications that may arise from suspending the antitachycardia functionality of the AICD, respectively.

## Conclusions

In conclusion, we have presented a case that followed the 2020 ASA practice advisory for perioperative CIED management, but the AICD defibrillated twice. Thus, when monopolar electrocautery will be used, we encourage anesthesia personnel to evaluate not only the patient, the device interrogation report, whether the patient is currently pacemaker dependent, and the location of the surgery, but also EDP placement. Most guidelines regarding the perioperative management of CIEDs focus on above the umbilicus procedures, demonstrating the need for more definitive guidance with below the umbilicus operations. Literature regarding EDP placement specifically in patients with CIEDs remains scarce, if not non-existent, given the mere theoretical risk EDP radiofrequency dispersion holds. Further research should determine best practices specifically for perioperative CIED management in surgeries below the umbilicus with limited options for EDP placement.
